# 5-year clinical and radiographic follow-up of the uncemented Symax hip stem in an international study

**DOI:** 10.1186/s13018-018-0888-9

**Published:** 2018-07-31

**Authors:** Dennis Silvester Maria Gerardus Kruijntjens, Per Kjaersgaard-Andersen, Peter Revald, Jane Schwartz Leonhardt, Jacobus Johannes Chris Arts, René Hendrikus Maria ten Broeke

**Affiliations:** 1 Department of Orthopaedic Surgery, Research School Caphri, Maastricht University Medical Centre, P. Debyelaan 25, P.O. Box 5800, 6202 AZ Maastricht, the Netherlands; 2 Department of Orthopaedic Surgery, Vejle Hospital, Beriderbakken 4, 7100 Vejle, Denmark

**Keywords:** Symax, Uncemented hip stem, Clinical, Radiographic, Total hip arthroplasty

## Abstract

**Background:**

The uncemented Symax hip stem is developed through optimization of the uncemented Omnifit hip stem. The Symax stem design combines an anatomical anteverted proximal geometry with a straight distal section. The proximal part is coated with a biomimetic hydroxyapatite (HA) coating for improved osseointegration to enhance load transfer and to minimize proximal bone loss. The distal part is treated with an anodization surface treatment in order to prevent distal bone apposition, which is expected to prevent distal loading and reduce proximal stress shielding. Aim of this study is to report mid-term clinical performance and evaluate whether the radiographic features are in line with the design principles of the Symax hip.

**Methods:**

The biomimetic hydroxyapatite-coated uncemented Symax hip stem was evaluated in 80 patients during a 5-year prospective clinical international study. Harris Hip Score (HHS), Oxford Hip Score (OHS), and Western Ontario and McMaster Universities Arthritis Index (WOMAC) were performed preoperatively and postoperatively at 6 months and 1, 2, 3 and 5 years. Anteroposterior radiographs of the pelvis and axial radiographs of the operated hips were evaluated immediately postoperative and at follow-up 6 months and 1, 2, 3, and 5 years. Wilcoxon signed-rank test was used to analyse whether clinical outcome scores changed statistically significant over time. The overall percentage of agreement between two radiology assessment teams was used to evaluate observer agreement of radiology results. The Cohen’s Kappa was evaluated as a measure of reliability to quantify the agreement between raters, corrected for chance agreement.

**Results:**

Clinical outcome scores were excellent at 5 years with mean HHS of 98.1, mean OHS of 16.2 and mean WOMAC of 6.9. Only 2.7% of the patients had pain at rest or on weight-bearing, and mid-thigh pain was reported by 1.4% of the patients after 5 years. The percentage of agreement between radiology assessment teams was 94 to 100%, except for distal line formation (48%). Radiographic evaluation showed stable stems and signs of excellent progressive proximal fixation and favourable bone remodeling.

**Conclusions:**

The excellent mid-term clinical and radiographic performances are in line with the design principles and coating properties of this new implant and earlier published results.

**Trial registration:**

http://ClinicalTrials.gov, NCT03469687. Registered 19 March 2018 – Retrospectively registered.

## Background

New designs of uncemented hip stems focus on enhancing osseointegration, improved interface sealing, optimized load transfer, diminishing the rate of loosening, and on improving clinical outcomes. For these purposes, optimizations can be realized in the geometry of the stem, the choice of material, surface texture, and the type and extent of the osseointegrative coating [1–6]. These considerations resulted in the development of the uncemented Symax stem design (Stryker Orthopaedics, Amsterdam, the Netherlands) as an optimization of the well-documented, second-generation uncemented Omnifit hip stem [3, 7, 8]. Histological and histomorphometric analyses on retrieved implanted Symax hip stems have already proven early proximal ingrowth as a result of the new BONIT-hydroxyapatite (HA) coating (DOT GmbH, Rostock, Germany) and the distal DOTIZE surface treatment (DOT GmbH, Rostock, Germany) [9]. Furthermore, improved bone remodeling was already established in a 2-year follow-up dual-energy X-ray absorptiometry (DEXA) study compared to the Omnifit hip stem [10]. This international study is part of a stepwise clinical introduction of the Symax hip stem according to Malchau et al. [11], illustrating the ‘phased innovation’ of this new implant. As part of this clinical introduction also, a prospective radiostereometric analysis (RSA) study and a large prospective clinical cohort study are ongoing. The aim of this study is to report mid-term clinical performance and evaluate whether the radiographic features are in line with the design principles of the Symax hip stem in an international setting during 5 years of clinical follow-up in a cohort of 80 patients.

It was hypothesized that the new BONIT-HA coating and the distal DOTIZE surface treatment, together with an optimized geometrical design, would generate both a mechanically stable stem with mid-term radiographic features of consistent and progressive excellent proximal fixation and radiographic signs that would also underline the effects of the distal surface treatment. The combination of these would anticipate a superior bone remodeling that is highly recognizable on conventional radiographs.

## Methods

Between September 2004 and November 2005, 80 patients were included in this prospective international study performed at the Maastricht University Medical Centre (MUMC), the Netherlands (centre 1, *n* = 30), and the Vejle Hospital, Denmark (centre 2, *n* = 50). Eligibility criteria were patients requiring primary uncemented total hip arthroplasty (THA), age older than 18 years, and BMI less or equal to 35. Exclusion criteria were bilateral hip complaints, impaired cognitive function, and use of medication or illness influencing bone metabolism. Baseline demographic data were similar for both study centres (Table 1).

**Table 1:** Baseline demographic data of study cohort

	Centre 1	Centre 2	Total	*P* value
No. of hips	30	50	80	
Male/female	16/14	29/21	45/35	0.684
Mean (range) age (years)	57.5 (41–71)	56.2 (30–69)	56.7 (30–71)	0.437
Mean (range) BMI (kg/m^2^)	27.1 (20–33)	27.0 (18–35)	27.0 (18–35)	0.913
Diagnosis, no. of hips (%)				0.135
Osteoarthritis	27 (90.0%)	41 (82.0%)	68 (85.0%)	
Avascular necrosis	2 (6.7%)	1 (2.0%)	3 (3.8%)	
Posttraumatic arthritis		7 (14.0%)	7 (8.8%)	
Other	1 (3.3%)	1 (2.0%)	2 (2.5%)	

Ethical board approval was obtained from the local Institutional Review Board (centre 1: METC 04-112; centre 2: S-VF-20040133), and informed consent was obtained from each patient prior to surgery. This study was conducted according to the ethical standards of the Declaration of Helsinki of 1975, as revised in 2013 in Fortaleza (Brasil), and following the ISO 14155 Good Clinical Practice (GCP) guidelines.

### Surgical protocol

The posterolateral approach to the hip was used by four senior hip surgeons. Patients received 24-h intravenous antibiotic prophylaxis and deep venous thrombosis prophylaxis with low molecular weight heparins. Full weight-bearing was allowed from the first postoperative day. In this study, complete excision of the joint capsule was performed in 86.7% of the patients in centre 1 compared to 2.0% in centre 2. Non-steroidal anti-inflammatory drug (NSAIDs) prescription to prevent heterotopic bone formation also varied; 96.7% of the patients in centre 1 received NSAIDs, compared to only 4.0% of patients in centre 2.

### Implant

**Fig. 1:**
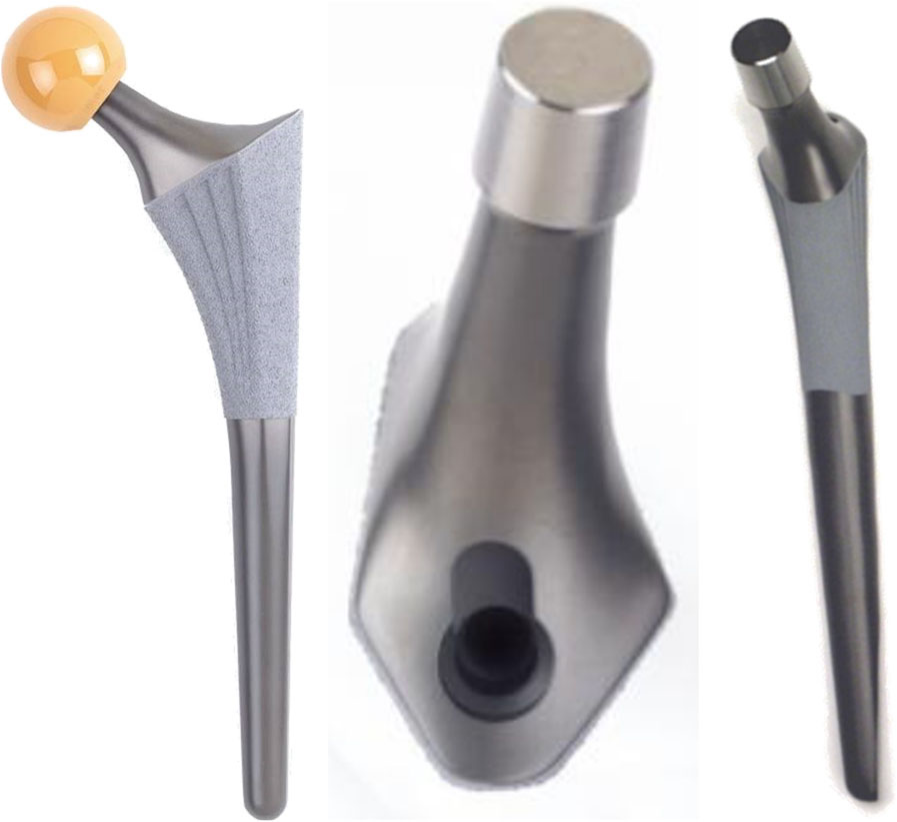
Design features of Symax hip stem, illustrating the anatomically anteverted proximal geometry and the distal posterior chamfer

The Symax hip stem is an uncemented design forged from Ti6Al4V alloy. Primary mechanical stability is provided by anatomical metaphyseal geometry, based on CT-analysis of the proximal femur (data on file at Stryker). The hip stem features a size-dependent anteversion, neck length, and offset, with a centrum-collum-diaphyseal (CCD) angle of 128° (data on file at Stryker). Axial stability is pursued by the straight distal section in the femoral canal (Fig. 1). Secondary biological stability is accomplished by fast osseous integration due to the BONIT-HA coating on the metaphyseal part of the stem (Fig. 1), as was confirmed earlier by histology and histomorphometry analyses on retrieved stems [9]. BONIT-HA is a new generation, electrochemically deposited, biomimetic hydroxyapatite (HA) coating on top of a commercially pure titanium plasma spray (TPS) layer. It is deposited by low-temperature precipitation, is thin (10–20 μm), and has a 3D surface with high porosity (60%) and pore interconnectivity [9, 10, 12, 13]. The coating is fully resorbable and is known to be substituted by bone for about 99% [14]. The anodization surface treatment, DOTIZE, applied on the distal part of the stem, is an electrolytical conversion of the native oxide film on titanium surfaces into a thicker and denser titanium oxide. It shows anti-galling properties and reduces protein adsorption with 19% and bone apposition compared to untreated titanium alloy [9, 10, 15].

The uncemented HA-coated Trident acetabular cup (Stryker Orthopaedics, Mahwah, NJ, USA) was used in 78 patients (98%) [16], except 2 patients (2.5%) in centre 1 who had cemented SHP ArCom ultra-high molecular weight polyethylene cups (Biomet, Bridgend, UK). Of the 78 uncemented cups, 75 patients (96%) had a ceramic insert (Alumina) and 3 patients (3.8%) had a highly cross-linked polyethylene insert. All patients had a ceramic head (Alumina). Head diameter of 32 mm was used in 52 patients (65%), 28 mm in 14 patients (18%), and 36 mm in 14 patients (18%).

### Clinical evaluations

Clinical evaluations were performed preoperatively and postoperatively at 6 months, 1 year, 2 years, 3 years, and 5 years. Evaluated clinical outcome parameters were a hip-specific functional score (Harris Hip Score (HHS)) [17], a patient-centred hip score (Oxford Hip Score (OHS)) [18], and a disease-specific quality-of-life outcome measure (Western Ontario and McMaster Universities Osteoarthritis Index (WOMAC)) [19]. Furthermore, the incidence of thigh pain and overall pain at rest and on weight-bearing was evaluated. The amount of pain was classified as no pain, slight, mild, moderate, marked, or totally disabling [17]. Complications and adverse events were recorded during follow-up, and the resulting survival analysis according to Kaplan-Meier was evaluated at the final follow-up.

### Radiographic evaluations

Anteroposterior radiographs of the pelvis and axial radiographs of the operated hips were evaluated immediately postoperative and at follow-up 6 months, 1 year, 2 years, 3 years, and 5 years. Migration was assessed according to the criteria of Malchau et al. [20]. Reactive lines, cancellous condensation (‘spotwelds’), cortical hypertrophy, and tip sclerosis were evaluated per Gruen zone [21]. Implant fixation and stability was assessed according to the modified Engh score [22]. Formation of heterotopic bone was evaluated using Brooker’s classification [23]. All radiographs were independently assessed by two teams of each two observers (PKA, DK and RtB, PR), consisting of one orthopaedic surgeon of both centres. Both teams of observers were blinded for each other’s assessment and the name and details of the patient, as well as for the time of follow-up of the radiographs. Furthermore, both teams evaluated 50 randomly chosen radiographs to determine agreement and to calculate intra-observer and inter-observer reliability. When 95% agreement was established, each team evaluated half of all radiographs of the total patient cohort.

### Statistics

SPSS for Windows 17.0.1 (SPSS Inc., Chicago, Illinois, USA) was used for statistical data analysis. Descriptive statistics were used for patient characteristics. Pearson’s chi-square test and Student’s *t* test were used to test hypotheses between both study centres for categorical variables and continuous variables respectively. Wilcoxon signed-rank test was used to analyse whether clinical outcome scores changed statistically significant over time. Statistical significance was set at *p* < 0.05. Results are reported as means, with standard deviation (SD) or range where relevant, or as frequencies. The overall percentage of agreement was used to evaluate observer agreement of radiology results [24]. The Cohen’s Kappa was evaluated as a measure of reliability to quantify the agreement between raters, corrected for chance agreement. Kaplan-Meier survival analysis was performed with revision for any reason and with revision of stem for aseptic loosening as the endpoints.

## Results

After 5 years of clinical follow-up, 73 patients were evaluated. Follow-up was not completed by one patient because of revision of both stem and cup for recurrent dislocations, one patient had a revision of the insert for squeaking, and five patients were lost to follow-up. One of these patients moved to another geographic area, the remaining four patients discontinued participation for non-hip-related reasons; one patient was from centre 1, and the remaining six patients from centre 2. Retrospective inquiry after a minimum 5-year follow-up learned that there were no hip-related complaints and no revision hip surgery was performed elsewhere.

### Clinical evaluations

Mean HHS improved from 58.9 ± 12.0 preoperatively to 98.1 ± 6.1 at 5-year clinical follow-up, mean OHS improved from 38.5 ± 6.0 preoperatively to 16.2 ± 6.4 after 5 years, and mean WOMAC improved from 48.2 ± 13.7 preoperatively to 6.9 ± 9.9 after 5 years of clinical follow-up (Table 2). All clinical outcomes showed statistically significant improvements between preoperative and 6 months postoperative scores (*p* < 0.001), which remained thereafter.

**Table 2:** Mean clinical outcome parameters

	Preoperative	6 months	1 year	2 years	3 years	5 years
Mean HHS (± SD)	58.9 (12.0)	94.3 (9.1)	95.2 (10.5)	96.1 (8.7)	97.4 (7.7)	98.1 (6.1)
Wilcoxon (*P* value)		< 0.001	0.074	0.121	0.015	0.482
Mean OHS (± SD)	38.5 (6.0)	20.0 (9.4)	18.7 (8.1)	17.4 (7.7)	16.0 (7.1)	16.2 (6.4)
Wilcoxon (*P* value)		< 0.001	0.028	0.072	0.012	0.081
Mean WOMAC (± SD)	48.2 (13.7)	13.0 (14.5)	10.8 (13.7)	9.7 (13.4)	9.0 (13.7)	6.9 (9.9)
Wilcoxon (*P* value)		< 0.001	0.019	0.673	0.181	0.125

Mean HHS, OHS, and WOMAC with standard deviation (SD) and Wilcoxon ranked sign test evaluating improvement over time between two consecutive follow-up moments

After 5 years, one patient (1.4%) complained of moderate lateral thigh pain at rest and on weight-bearing. No radiographic abnormalities could be detected for this patient. During follow-up, the number of patients with no, or slight, pain at rest improved from 21.3% preoperatively to 97.3% at 5 years. The number of patients with no, or slight, pain on weight bearing also improved from 2.5% preoperatively to 97.3% at 5 years.

### Adverse events

During primary surgery, two patients developed an acetabular fracture for which intraoperative interventions were performed. These patients were not allowed immediate full weight-bearing postoperatively. Four patients had early dislocations, three of them were successfully treated conservatively and the fourth patient underwent revision of both cup and stem for a design with more femoral offset. Conservative treatment of early dislocations in our centres consisted of giving patients more instructions about prohibited movements of deep flexion and internal rotation, and we referred the patients back to their physiotherapists. One patient developed an early deep infection with *Staphylococcus aureus* 1 month after primary surgery. This patient was successfully treated with extensive debridement and with local and systemic antibiotic therapy. The implant could be retained. Finally, one patient complained of squeaking for which revision of the ceramic insert to a highly cross-linked polyethylene insert (X3, Stryker Orthopaedics) was performed.

**Fig. 2:**
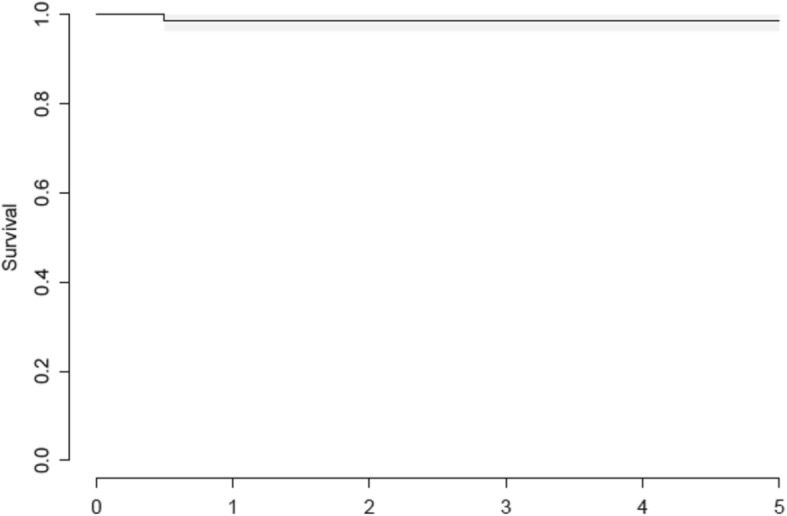
Kaplan-Meier curve for revision of stem for any reason, in years

Survival of the Symax hip stem after 5 years with revision of the stem for any reason as the endpoint is 98.8%, as only 1 patient of the initial 80 patients had a revision of stem and cup for recurrent dislocations (Fig. 2). However, survival of the Symax stem is 100% after 5 years with revision of the stem for loosening as the endpoint.

### Radiographic evaluations

The percentage of agreement between both radiology assessment teams for spotweld formation was 94%, with a Cohen’s Kappa of 0.380. The percentage of agreement between both radiology assessment teams for other radiological evaluations was 100%, no Cohen’s Kappa could be measured for these evaluations. Only distal line formation could not be evaluated with high percentage of agreement, which was calculated at 48%, with a Cohen’s Kappa of 0.155.

**Fig. 3:**
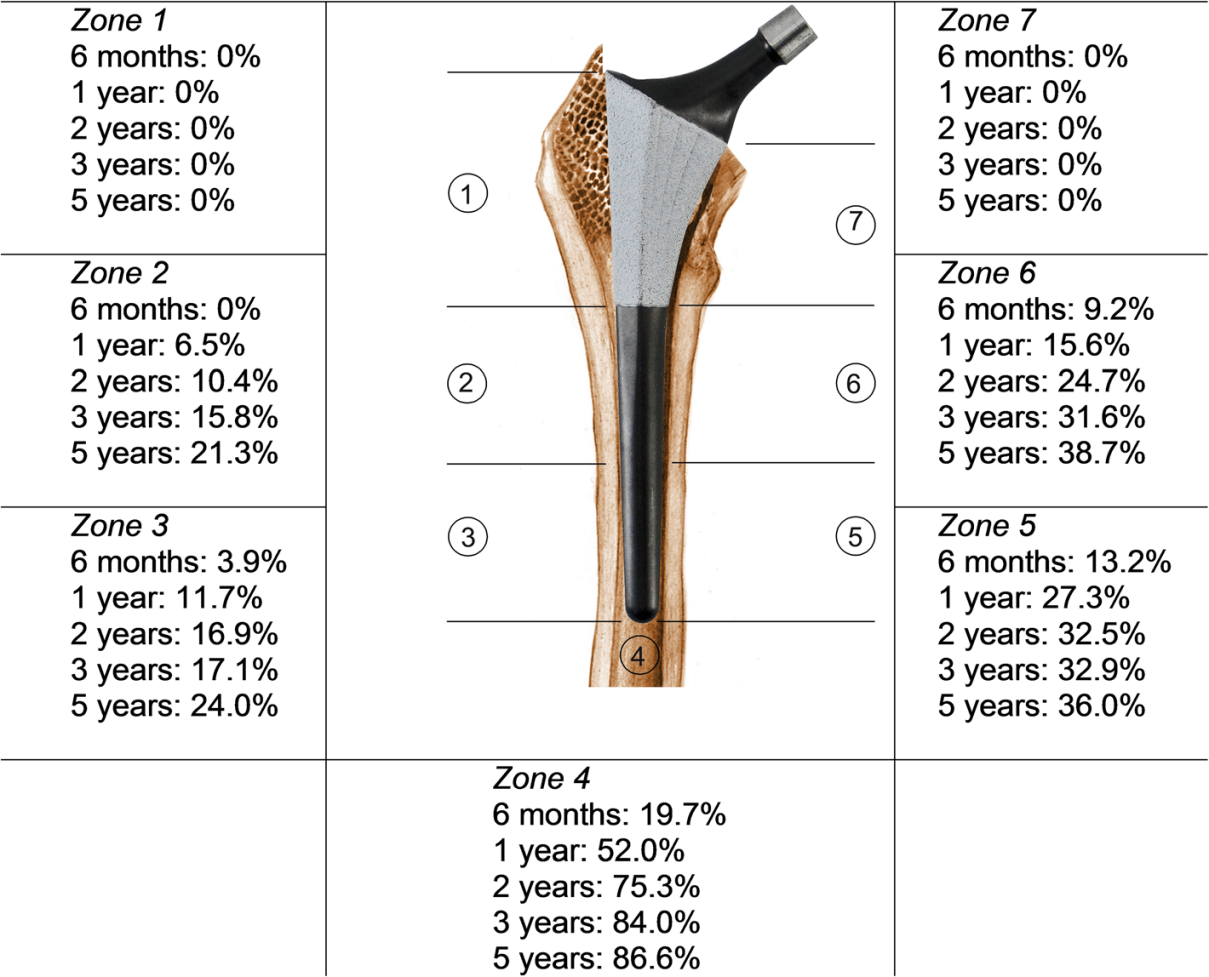
Outcomes of radiographic evaluation: reactive line formation

**Fig. 4:**
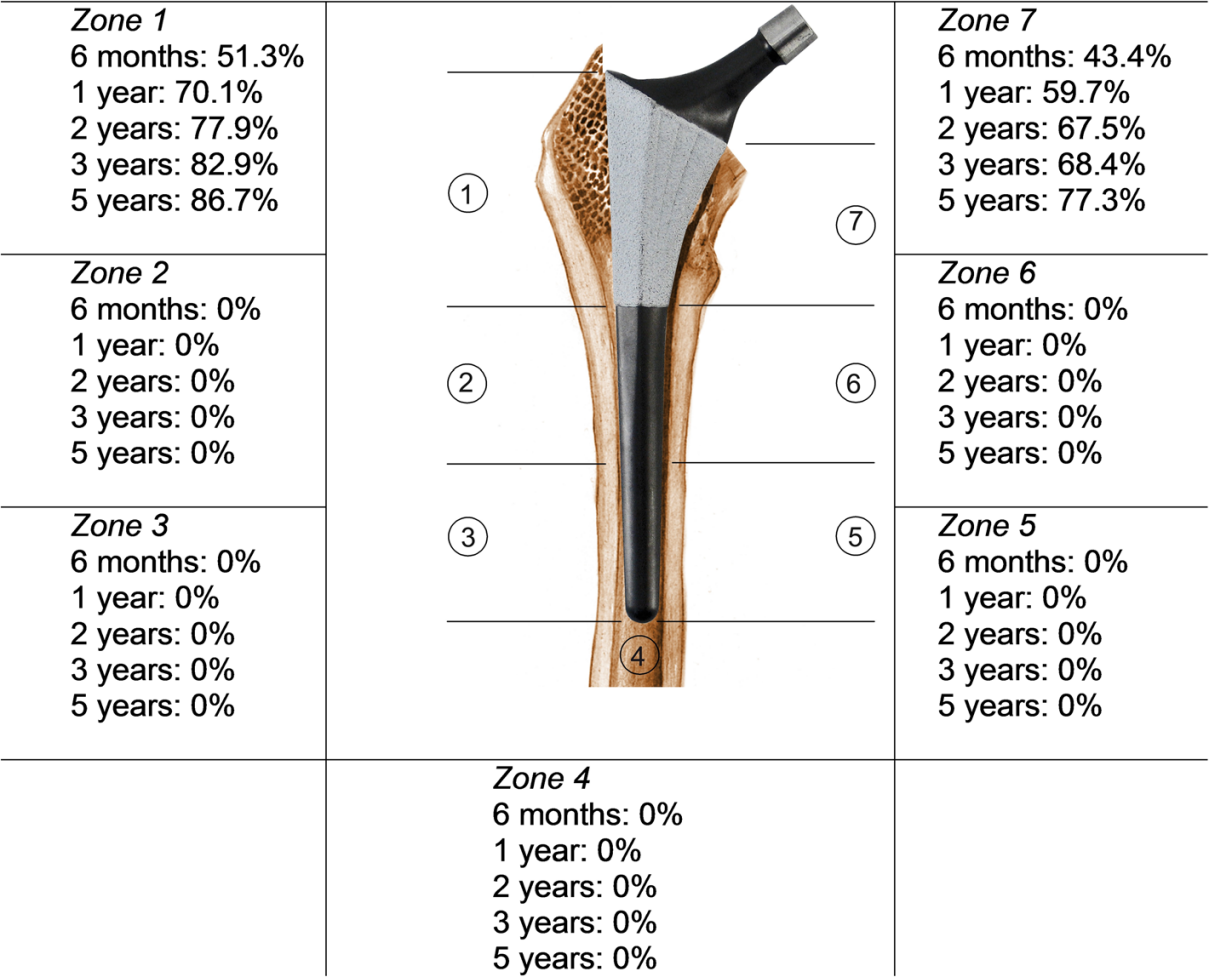
Outcomes of radiographic evaluation: cancellous densification (‘spotwelds’)

Stem position was neutral for all stems. No osteolysis or signs of proximal stress shielding were found during follow-up. One patient (1.3%) showed distal migration of the stem during follow-up. No lines or lucencies were observed around the coated part of the prosthesis at any follow-up time point. The appearance of extensive line formation around the smooth, uncoated, distal part of the stem increased from 10.5% after 6 months to 40% at 5 years. The appearance of distal line formation was most common in Gruen zone 4, 5, and 6 and increased during follow-up (Fig. 3). The appearance of spotwelds increased from 63.2% at 6 months to 90.7% at 5 years, only in the coated proximal Gruen zones 1 and 7 (Fig. 4). Any degree of heterotopic ossifications (Brooker 1 to 4) was seen in 29.7% of patients at 6 months; this number increased to 40.5% at 5 years. Only one patient (1.4%) showed Brooker 4 heterotopic ossification, which was already present at 6 months.

## Discussion

The purpose of this study was to evaluate clinical and radiographic performance of the new uncemented Symax hip stem in a 5-year international study, as part of the ‘phased innovation’ of a new implant, and to assess whether these outcomes were in line with the design principles and coating properties of the Symax hip stem. Our data showed excellent mid-term clinical and radiographic performances and osseointegration of the Symax hip stem at 5-year clinical follow-up with a Kaplan-Meier survival of 98.8% with revision of the stem for any reason as an endpoint.

The Symax hip stem leads to excellent clinical performance as represented in HHS, OHS, and WOMAC in this 5-year clinical follow-up study. Mean HHS of the Symax hip stem is slightly better compared to the short- and mid-term HHS of the Omnifit hip stem [3, 7, 8, 25, 26]. The Omnifit hip stem is another example of a proximal press fit, HA-coated stem, on which geometry the Symax was optimized. HHS of the Omnifit hip stem during the first 3 years of clinical follow-up was 93 after 6 months, improving to 96 at 3 years [26] and stabilizing to 93 after 6.4 years [27]. The clinical relevance of such small differences in HHS however can be argued as described by Poolman et al., who showed high intra-observer variability for physical examination as part of the HHS [28], and by Wamper et al. who showed limited discriminating power of the HHS because of a ceiling effect [29]. Nevertheless, the excellent HHS in this study illustrates state of the art performance of the Symax hip stem design.

According to Lieberman et al., we also conducted patient-oriented quality-of-life surveys, like the OHS and WOMAC [30, 31]. The OHS improved over time and led to excellent scores postoperatively [18, 32]. Mean WOMAC score showed the same pattern of improvement over time as OHS [19], confirming high patient satisfaction.

Another interesting observation of this study was that the absence of pain at rest and during weight-bearing. This was seen in 97.3% of the patients after 5 years, while only 1.4% of the patients had moderate lateral thigh pain. Although this result is only slightly better than other contemporary implants based on the same philosophy [8, 26, 27, 33–35], it is clearly superior to older designs that follow different philosophies [36, 37]. This may be related to the distal geometry with posterior chamfer to prevent stem tip impingement. Furthermore, the prevention of distal fixation will also prevent conflicting elasticity issues between the stiffer implant and the more flexible bone.

Longitudinal radiographic evaluations showed signs of enhanced proximal fixation with the consistent appearance of spotwelds in Gruen zones 1 and 7, while on the other hand, no radiolucencies or reactive lines were seen in the coated part of the stem. These signs of proximal fixation combined with increasing line formation around the distal uncoated part of the prosthesis can be explained by the BONIT-HA coating and DOTIZE surface treatment of the Symax hip stem. As illustrated from histomorphometry analyses of retrieval specimens, BONIT-HA coating results in osseointegration over a larger part of the coated stem surface [9, 14]. The highly bioactive biomimetic BONIT-HA coating results in both more extensive bone-implant contact and bone density in periprosthetic regions of interest compared to other currently known hydroxyapatite or porous-coated stems [9, 10]. Enhanced proximal fixation combined with reduced distal bone apposition, caused by the DOTIZE surface treatment, leaves space for relative ‘micromotion’ between the stiff distal stem and the more elastic bone [25]. This leads to a so-called ‘windshield-wiper sign’ [33], causing increasing occurrence of distal reactive lines around the Symax hip stem, which proves the theoretical concept of this implant design. With this, distal reactive line formation could be a useful radiographic sign of good proximal fixation. The measure of agreement for distal line formation was only 48% which suggests that this is not a reproducible and useful radiographic sign for evaluation. However, this can be explained by the subjective interpretation of the term ‘extensive’ distal line formation. A better and uniform definition of the term extensive distal line formation will probably result in a higher measurement of agreement. This makes this radiographic sign more useful for evaluation of proximally fixating uncemented hip stems. The high measure of agreement for spotweld formation and the absence of proximal line formation make them useful and reliable radiographic features for evaluating these kinds of stems radiographically over time. The measure of agreement is more useful to evaluate observer agreement of radiology results compared to Cohen’s Kappa, as it is not influenced by the prevalence of the evaluated radiographic signs [24]. So, the absence of proximal line formation in combination with progressive spotweld formation around the coated part and distal line formation should be interpreted as radiographic features of good proximal stem fixation.

Although 5 years of clinical follow-up is too short, a time frame to draw conclusions about final outcome and the survival rates of the Symax hip stem of 98.8%, with revision of the stem for any reason as the end point and with revision of the stem for (a)septic loosening as the end point of 100%, were excellent and meet the ‘entry benchmark’ criteria for best prostheses following the NICE-criteria [38]. These survival rates correspond to the survival rates of the Symax hip stem in the Danish Hip Arthroplasty Registry (DHR) of 2013 [39]. In the DHR, the Symax hip stem has a survival rate of 100% after 5 years with revision for aseptic loosening. The survival rate with revision for any reason is 97.7 to 98.1%, depending on the cup used, after 5 years [39]. Based on what is known from other older HA-coated stems, good long-term fixation therefore can be anticipated.

This study had some limitations. Although it was well protocolized regarding surgical technique and postoperative treatment, some minor differences in treatment protocol existed between the two centres that can be interpreted as limitations. Complete excision of the joint capsule during the inclusion period of this study was only predominantly performed in centre 1. Surprisingly, capsulectomy did not seem to influence the occurrence of dislocations in this study, as both centres had two patients with dislocations. There was no difference in the amount of heterotopic bone formation between both study centres, despite the difference in strategies for prevention of it. Some authors have reported a decrease in osseointegration of implants as a result of the use of NSAIDs [40–42], and a non-significant increase in implant loosening [43]. However, in line with other reports [44–46], we could not find signs of a difference in bony fixation on conventional radiography between the two centres nor was there more pain reported in one or either centre.

Compared to previous literature of the Symax hip stem, the fact that only one patient showed migration, but no loosening, was in line with the EBRA-FCA study by Buratti et al. [47]. Our results are also in line with the findings of good clinical performance of this implant as was reported in a 1 year prospective study, in which the Symax was compared to the predominantly diaphyseal anchored Hipstar hip stem (Stryker, Duisburg, Germany) and the straight Zweymuller (SL-Plus) hip stem (Plus Orthopedics AG, Rotkreuz, Switzerland) [48]. However, Bergschmidt et al. discontinued using the Symax hip stem because of subsidence of more than 10 mm in two patients and three intraoperative periprosthetic fractures outside the study group. We cannot confirm these complication frequencies in this current study population nor in a multicentre prospective cohort of 300 patients. We therefore do not believe this to be a prosthesis-related problem.

## Conclusions

In summary, excellent mid-term clinical and radiographic performance of the Symax hip stem can be reported at 5-year clinical follow-up. This is in line with the design principles and coating properties of this new stem design. Radiographic features of bone remodeling and (proximal) stem fixation around this design show high overall percentage agreement for intra- and inter-observer reliability, making them useful tools for longitudinal follow-up. In view of already available histological and remodeling data, good long-term performance of the Symax hip stem may be anticipated. Further confirmation of long-term result is subject of already initiated follow-up studies of both national and international prospective cohorts.

## Abbreviations

BMI: Body mass index
CCD: Centrum-collum-diaphyseal
DEXA: Dual-energy X-ray absorptiometry
GCP: Good Clinical Practice
HA: Hydroxyapatite
HHS: Harris Hip Score
MUMC: Maastricht University Medical Centre
NSAID: Non-steroidal anti-inflammatory drugs
OHS: Oxford Hip Score
RSA: Radiostereometric analysis
SD: Standard deviation
THA: Total hip arthroplasty
TPS: Titanium plasma spray
WOMAC: Western Ontario and McMaster Universities Arthritis Index

## References

[1] Bobyn JD, Glassman AH, Goto H, Krugier JJ, Miller JE, Brooks LE. The effect of stem stiffness on femoral bone resorption after canine porous-coated total hip arthroplasty. Clin Orthop Relat Res. 1990;(261):196–213. 2245546

[2] Bobyn JD, Mortimer ES, Glassman AH, Engh CA, Miller JE, Brooks LE (1992). Producing and avoiding stress shielding. Laboratory and clinical observations of noncemented total hip arthroplasty. Clin Orthop Relat Res.

[3] Capello WN, D'Antonio JA, Geesink RG, Feinberg JR, Naughton M (2009). Late remodeling around a proximally HA-coated tapered titanium femoral component. Clin Orthop Relat Res.

[4] Engh CA, Bobyn JD (1988). The influence of stem size and extent of porous coating on femoral bone resorption after primary cementless hip arthroplasty. Clin Orthop Relat Res.

[5] Glassman AH, Bobyn JD, Tanzer M (2006). New femoral designs: do they influence stress shielding?. Clin Orthop Relat Res.

[6] Huiskes R, Weinans H, Dalstra M (1989). Adaptive bone remodeling and biomechanical design considerations for noncemented total hip arthroplasty. Orthopedics.

[7] Capello WN, D'Antonio JA, Jaffe WL, Geesink RG, Manley MT, Feinberg JR (2006). Hydroxyapatite-coated femoral components: 15-year minimum followup. Clin Orthop Relat Res.

[8] Hellman EJ, Capello WN, Feinberg JR (1999). Omnifit cementless total hip arthroplasty. A 10-year average followup. Clin Orthop Relat Res.

[9] Broeke ten RHM, Alves A, Baumann A, Arts JJC, Geesink RGT (2011). Bone reaction to a biomimetic third-generation hydroxyapatite coating and new surface treatment for the Symax hip stem. J Bone Joint Surg (Br).

[10] Broeke ten RH, Hendrickx RP, Leffers P, Jutten LM, Geesink RG (2012). Randomised trial comparing bone remodelling around two uncemented stems using modified Gruen zones. Hip Int..

[11] Malchau H, Bragdon CR, Muratoglu OK (2011). The stepwise introduction of innovation into orthopedic surgery: The next level of dilemmas. J Arthroplasty.

[12] Barrere F, Layrolle P, Van Blitterswijk CA, De Groot K (2001). Biomimetic coatings on titanium: a crystal growth study of octacalcium phosphate. J Mater Sci Mater Med.

[13] Becker P, Neumann HG, Nebe B, Luthen F, Rychly J (2004). Cellular investigations on electrochemically deposited calcium phosphate composites. J Mater Sci Mater Med..

[14] Szmukler-Moncler S, Perrin D, Ahossi V, Pointaire P (2001). Evaluation of BONIT, a fully resorbable CaP coating obtained by electrochemical deposition, after 6 weeks of healing: a pilot study in the pig maxilla. Key Eng Mater.

[15] Becker P, Baumann A, Lüthen F, Rychly J, Kirbs A, Beck U, et al. Spark anodization on titanium and titanium alloys. 10th World Conference on Titanium. Hamburg, Germany 2003;V:3339–3344.

[16] D'Antonio JA, Capello WN, Manley MT, Naughton M, Sutton K (2005). A titanium-encased alumina ceramic bearing for total hip arthroplasty: 3- to 5-year results. Clin Orthop Relat Res.

[17] Harris WH (1969). Traumatic arthritis of the hip after dislocation and acetabular fractures: treatment by mold arthroplasty. An end-result study using a new method of result evaluation. J Bone Joint Surg Am.

[18] Dawson J, Fitzpatrick R, Carr A, Murray D (1996). Questionnaire on the perceptions of patients about total hip replacement. J Bone Joint Surg (Br).

[19] Bellamy N, Buchanan WW, Goldsmith CH, Campbell J, Stitt LW (1988). Validation study of WOMAC: a health status instrument for measuring clinically important patient relevant outcomes to antirheumatic drug therapy in patients with osteoarthritis of the hip or knee. J Rheumatol.

[20] Malchau H, Karrholm J, Wang YX, Herberts P (1995). Accuracy of migration analysis in hip arthroplasty. Digitized and conventional radiography, compared to radiostereometry in 51 patients. Acta Orthop Scand.

[21] Gruen TA, McNeice GM, Amstutz HC (1979). “Modes of failure” of cemented stem-type femoral components: a radiographic analysis of loosening. Clin Orthop Relat Res.

[22] Engh CA, Massin P, Suthers KE (1990). Roentgenographic assessment of the biologic fixation of porous-surfaced femoral components. Clin Orthop Relat Res.

[23] Brooker AF, Bowerman JW, Robinson RA, Riley LH (1973). Ectopic ossification following total hip replacement. Incidence and a method of classification. J Bone Joint Surg (Am).

[24] Vet de HC, Mokkink LB, Terwee CB, Hoekstra OS, Knol DL (2013). Clinicians are right not to like Cohen’s kappa. BMJ.

[25] D'Antonio JA, Capello WN, Crothers OD, Jaffe WL, Manley MT (1992). Early clinical experience with hydroxyapatite-coated femoral implants. J Bone Joint Surg Am.

[26] D'Antonio JA, Capello WN, Jaffe WL (1992). Hydroxylapatite-coated hip implants. Multicenter three-year clinical and roentgenographic results. Clin Orthop Relat Res.

[27] Capello WN, D'Antonio JA, Feinberg JR, Manley MT (1997). Hydroxyapatite-coated total hip femoral components in patients less than fifty years old. Clinical and radiographic results after five to eight years of follow-up. J Bone Joint Surg Am.

[28] Poolman RW, Swiontkowski MF, Fairbank JC, Schemitsch EH, Sprague S, Vet de HC (2009). Outcome instruments: rationale for their use. J Bone Joint Surg Am.

[29] Wamper KE, Sierevelt IN, Poolman RW, Bhandari M, Haverkamp D (2010). The Harris hip score: do ceiling effects limit its usefulness in orthopedics?. Acta Orthop.

[30] Lieberman JR, Dorey F, Shekelle P, Schumacher L, Kilgus DJ, Thomas BJ (1997). Outcome after total hip arthroplasty. Comparison of a traditional disease-specific and a quality-of-life measurement of outcome. J Arthroplasty.

[31] Lieberman JR, Dorey F, Shekelle P, Schumacher L, Thomas BJ, Kilgus DJ (1996). Differences between patients’ and physicians’ evaluations of outcome after total hip arthroplasty. J Bone Joint Surg Am.

[32] Ashby E, Grocott MP, Haddad FS (2008). Outcome measures for orthopaedic interventions on the hip. J Bone Joint Surg (Br).

[33] Geesink RG, Hoefnagels NH (1995). Six-year results of hydroxyapatite-coated total hip replacement. J Bone Joint Surg (Br).

[34] Incavo SJ, Havener T, Benson E, McGrory BJ, Coughlin KM, Beynnon BD (2004). Efforts to improve cementless femoral stems in THR: 2- to 5-year follow-up of a high-offset femoral stem with distal stem modification (Secur-Fit Plus). J Arthroplast.

[35] Tonino AJ, Rahmy AI (2000). The hydroxyapatite-ABG hip system: 5- to 7-year results from an international multicentre study. The International ABG Study Group. J Arthroplasty.

[36] Hwang SK, Park JS (1995). Cementless total hip arthroplasty with AML, PCA and HGP prostheses. Int Orthop.

[37] Garcia-Cimbrelo E, Cruz-Pardos A, Madero R, Ortega-Andreu M (2003). Total hip arthroplasty with use of the cementless Zweymuller Alloclassic system. A ten to thirteen-year follow-up study. J Bone Joint Surg (Am).

[38] NHS. Guidance on the Selection of Prostheses for Primary Total Hip Replacement (2000). Technology appraisals guidance.

[39] Dansk Hoftealloplastik Register. Årsrapport. 2013. http://danskhoftealloplastikregister.dk/wp-content/uploads/2015/11/DHR-årsrapport-2013.pdf. Accessed 4 Apr 2017.

[40] Abdul-Hadi O, Parvizi J, Austin MS, Viscusi E, Einhorn T (2009). Nonsteroidal anti-inflammatory drugs in orthopaedics. J Bone Joint Surg Am.

[41] Dahners LE, Mullis BH (2004). Effects of nonsteroidal anti-inflammatory drugs on bone formation and soft-tissue healing. J Am Acad Orthop Surg.

[42] Trancik T, Mills W, Vinson N (1989). The effect of indomethacin, aspirin, and ibuprofen on bone ingrowth into a porous-coated implant. Clin Orthop Relat Res.

[43] Persson PE, Nilsson OS, Berggren AM (2005). Do non-steroidal anti-inflammatory drugs cause endoprosthetic loosening? A 10-year follow-up of a randomized trial on ibuprofen for prevention of heterotopic ossification after hip arthroplasty. Acta Orthop.

[44] Kjaersgaard-Andersen P, Schmidt SA (1991). Total hip arthroplasty. The role of antiinflammatory medications in the prevention of heterotopic ossification. Clin Orthop Relat Res.

[45] Wurnig C, Schwameis E, Bitzan P, Kainberger F (1999). Six-year results of a cementless stem with prophylaxis against heterotopic bone. Clin Orthop Relat Res.

[46] Heide van der HJ, Hannink G, Buma P, Schreurs BW (2008). No effect of ketoprofen and meloxicam on bone graft ingrowth: a bone chamber study in goats. Acta Orthop.

[47] Buratti CA, D'Arrigo C, Guido G, Lenzi F, Logroscino GD, Magliocchetti G (2009). Assessment of the initial stability of the Symax femoral stem with EBRA-FCA: a multicentric study of 85 cases. Hip Int.

[48] Bergschmidt P, Bader R, Finze S, Gankovych A, Kundt G, Mittelmeier W (2010). Cementless total hip replacement: a prospective clinical study of the early functional and radiological outcomes of three different hip stems. Arch Orthop Trauma Surg.

